# On-Call Duties: The Perceived Impact on Veterinarians' Job Satisfaction, Well-Being and Personal Relationships

**DOI:** 10.3389/fvets.2021.740852

**Published:** 2021-10-27

**Authors:** Lori Kogan, Regina Schoenfeld-Tacher, Patrick Carney, Peter Hellyer, Mark Rishniw

**Affiliations:** ^1^College of Veterinary Medicine and Biomedical Sciences, Colorado State University, Fort Collins, CO, United States; ^2^College of Veterinary Medicine, North Carolina State University, Raleigh, NC, United States; ^3^Community Practice Service, Cornell University College of Veterinary Medicine, Ithaca, NY, United States; ^4^Veterinary Information Network, Davis, CA, United States

**Keywords:** occupational health and safety, one health, public health and regulatory medicine, economics, quality of life

## Abstract

**Objective:** To assess the impact of on-call duties on veterinarians' job satisfaction, well-being and personal relationships.

**Design:** Cross-sectional survey.

**Sample:** The sample was obtained from Veterinary Information Network (VIN) members in private practice within the United States.

**Procedures:** A link to an anonymous online survey was distributed via an email invitation to all Veterinary Information Network (VIN) members with access from August 15, 2017 to October 21, 2017.

**Results:** A total of 1,945 responses were recorded. The majority of those who reported having on-call duties were female associates. Composite scales were created to assess the impact of on-call shifts on job satisfaction and well-being. Multiple linear regression was conducted and found that gender (*p* = 0.0311), associate status (*p* < 0.0001), and age (*p* = 0.0293) were all significantly associated with on-call related job satisfaction. Additionally, multiple linear regression found that gender (*p* = 0.0039), associate status (*p* < 0.0057), and age (*p* < 0.0001) were all significantly associated with on-call related well-being. On-call shifts were reported by many to have a negative impact on job satisfaction and well-being; this was especially pronounced for female associates. Females had on-call related job satisfaction scores that were, on average, 1.27 points lower than that of males (lower scores equates to lower job satisfaction). Further, females' average on-call related well-being scores were 1.15 points higher than that of males (lower scores equates to higher well-being).

**Conclusions and Clinical Relevance:** This study suggests that on-call shifts have a negative impact on veterinarian job satisfaction, well-being and personal relationships. The negative impact on job satisfaction and well-being is greatest for female associates. Veterinary medicine has been identified as a stressful occupation that can lead to psychological distress. It is therefore important to critically assess current practices that appear to increase stress and reduce emotional well-being. For this reason, it is suggested that veterinary hospitals explore alternative options to traditional on-call shifts.

## Introduction

On-call work is defined as times during which employees have to be available to be called into work if needed (1). Many medical professionals, including veterinary clinicians, are required to do offsite on-call work (where they do not have to remain at the workplace but have to respond if called) as part of their job (2). These shifts are typically defined as non-restrictive, meaning they allow the employee to use this time for their own purposes, and are therefore, legally considered rest time (government publishing office). Typically these shifts require employees to carry a phone and return to work within a set period of time (e.g., 30 min) if needed. On-call shifts are often scheduled in between regular working shifts and frequently include weekends and overnight (1, 3). On-call shifts are often used to provide 24/7 h daily coverage for facilities where emergencies require trained onsite personnel but the business volume does not require regular shift coverage (4). Many veterinary hospitals pay veterinarians and veterinary support staff a stipend to be on-call, a solution that many employers use to provide full shift coverage during off-peak hours for less money than a regularly staffed shift (5).

Numerous studies have illuminated many negative physical and psychological effects of being on-call (6–9). Part of what makes on-call work challenging is that it often combines both long work hours and night shifts. Long work hours have been shown to increase stress levels and burnout (10), reduce quality of life (11), decrease job satisfaction (12), and increase risk of physical health problems (13) and increase the use of alcohol (14). On-call shifts have been found to be associated with anxiety and depression (15), stress levels (16), and lower mood (17).

Part of these effects are likely due to the fact that on-call shifts can lead to circadian misalignment and poorer sleep (18). Physicians' on-call shifts are associated with sleep deprivation that increases daytime sleepiness (8, 19). This is problematic since sleep deprivation and fatigue have been shown to impair human performance (20). Williams (21), for example, found that sensorimotor and cognitive processing is faster during routine, daytime shifts when compared to overnight on-call shifts. Sleep problems and fatigue also pose risks to medical professionals' own safety as well as patient care (9, 22). On-call duties have been associated with increased risk of occupational injuries, medical errors (20, 21, 23) and decreased clinical performance—due in part to decreased visual memory, reaction times, vigilance, and overall cognitive abilities (24–28). Furthermore, recovery from these shifts is challenging; Wali (29) found that obtaining adequate sleep during non-call days does not sufficiently compensate for the sleep debt caused by on-call shifts.

Given these effects, it is not surprising that being on-call is inversely related to job satisfaction. On-call shifts have been found to be a major source of stress, depression, and job dissatisfaction among physicians and nurses (30–33), contributing to a burnout rate of 25–60% of physicians (34). Burnout rate averages 55% for veterinarians in the first 5 years after graduation, with higher rates for females than males (35, 36). In addition to personal stress, being on-call has been shown to have a negative impact on physicians' family life, causing work-home interference, communication challenges, increased household responsibility load on other family members, as well as decreased time with partners and children (37–39).

To counteract the stressors of work, it is important that employees have adequate recovery time (e.g., leisure time) away from work (40). The effort-recovery model (41) suggests that chronic incomplete recovery from work can lead to impaired physical and mental health (42–44). Being on-call can impact recovery from work for several reasons, including a negative impact on sleep and daily schedules. This can be even more challenging for women who often have more household and childcare duties than men (45, 46).

For all these reasons, it is imperative that the veterinary field explore the impact of on-call shifts on veterinarians, whether they are veterinary associates or practice owners. Given the fact that 80.5% of US veterinary students enrolled in 2017 were female, and over 60% of currently practicing US veterinarians are women (47), this study is timely. It is the first to explore the impact of on-call shifts on veterinarians. It is hypothesized that on-call shifts have negative impacts on job satisfaction, well-being and personal relationships. More specifically, however, it is hypothesized that on-call shifts negatively affect those with the least societal control and power. Therefore, we tested the hypothesis that female associates would report higher perceived negative impact in the areas of job satisfaction, well-being and personal relationships when compared to male associates, female owners and male owners.

## Methodology

An anonymous online survey was created to evaluate veterinarians' experiences and perceptions regarding on-call shifts and their impact on job satisfaction, well-being, and personal relationships. The survey was created and tested by researchers at Colorado State University and Veterinary Information Network (VIN). This study was approved by Colorado State University Institutional Review Board. After the survey was created, it was piloted by a small sample of VIN members. They tested the survey for appropriate branching and potentially ambiguous or missing response options. Relevant feedback was incorporated into the final version of the survey. A link to the survey was distributed via an email invitation to all VIN members (*n* ~ 35,000), and access was made available from August 15, 2017 to October 21, 2017. A reminder email was sent 2 weeks after the initial invitation. US VIN members practice in all 50 US states, have an average age of 45.5 and are ~69% female. Only data from respondents who stated they currently practice veterinary medicine in the US were included in the study. The study was categorized as exempt by Colorado State University's Institutional Review Board. The survey was administered directly via the VIN data collection portal, and branching logic was used to display only questions relevant to each participant. The first two questions were screening tools to ensure respondents were currently practicing veterinary medicine and had on-call responsibilities at some point in their career. Veterinarians who self-identified as not currently practicing or had never had on-call shifts were eliminated from further analysis. The body of the survey consisted primarily of Likert questions, for which participants were able to select their agreement with several statements regarding job satisfaction, personal well-being and personal relationships. Free-text boxes were provided for participants to enter brief alternative answers when none of the listed options applied to them. A final question at the end of the survey allowed for free-text entry of any comments participants chose to make about being on-call.

### On-Call Survey Results

Statistical methods: Summary statistics were calculated for all variables, including proportions for dichotomous variables, category proportions for polytomous variables, and means plus or minus standard deviations or medians and interquartile ranges for continuous variables. Normality of continuous variables was assessed via visual inspection of histograms coupled with evaluation of the Shapiro Wilk test. For Likert-based variables, the responses were recoded 1–5 for creation of composite scores.

Three composite scores were calculated based on the Likert variables. The composite score for job satisfaction was comprised of eight items such as “I feel the extra pay I get from being on call makes the inconvenience worth it” and “I feel that having on call duties negatively impacts my job satisfaction.” The composite score for well-being was comprised of five statements including “I experience a great deal of anxiety while on call, regardless of whether I am called in or not” and “I feel unable to relax when I am on call.” Lastly, the relationship composite score was derived from two items: “I feel being on call negatively impacts my relationship with my partner” and “I feel being on call causes significant disruptions in my family/personal life.”

Questions where a higher level of agreement indicated a negative response/impact were subtracted from the composite scores.

For each composite score, respondents for whom data were missing for any component question were excluded from the analysis of that composite.

Multiple linear regression was performed to evaluate the relationship between gender, associate/owner status, and age category with each of the composite scores. Respondents who indicated a gender other than male or female were too few in number to include in the analysis. Age categories were decadal and entered as a continuous predictor. Normality was evaluated by visual inspection of normal QQ plots of residuals. Homoscedasticity was evaluated via visual inspection of residual vs. predicted value plots. If substantial departures from normality or homoscedasticity were detected, transformation of the outcome was performed (e.g., logarithmic, square root) and normality and homoscedasticity were again assessed; if transformation was unable to resolve the issue, bootstrapping with robust regression was performed and the standard error estimates were compared to those of the original linear regression; if the standard errors differed substantially, the bootstrapped estimates were used, whereas the linear regression estimates were used if the difference was minimal. Collinearity diagnostics (tolerance, variance inflation factor, and Eigenvalues) were assessed to ensure stability of the estimates.

Exploratory factor analysis was performed to examine the underlying construct of the Likert data using iterated principal factor analysis with orthogonal varimax rotation. Cronbach's alpha was calculated for the overall Likert survey instrument and for any latent factor identified through exploratory factor analysis, both for all complete surveys and for all surveys with responses to at least 80% of the Likert items, with missing data addressed via the mean replacement method. A Cronbach's alpha >0.7 was considered suggestive of acceptable internal consistency. All analysis was implemented in commercial statistical software (SAS 9.4, SAS Institute, Cary, NC). Statistical significance was set at *p* < 0.05.

## Results

General demographics: A total of 1,945 respondents completed at least a portion of the survey. Summary demographics are presented in Table 1. Almost half [931 (49.0%)] were age 40 or younger. A total of 1,199 (62.9%) had no children; of those with children, 96.6% had three or fewer, with two being the most common. The most common practice type was small animal general practice [1,294 (66.5%)], followed by mixed animal practice [233 (12.0%)], small animal specialty/referral [131 (6.7%)], and emergency [114 (5.9%)]; no other practice type represented more than 5% of the total. The majority of respondents [1,601 (83.0%)] were employed full-time. Two-thirds (1,299) were associates and one third were practice owners. Three hundred and twelve (16.5%) worked in emergency clinics.

**Table 1:** Participant demographics.

**Variable**	**Response options**	**Number (%)**
Practice type	Small animal general	1,294 (66.5)
	Emergency	114 (5.9)
	Small animal specialty	131 (6.7)
	Mixed animal	233 (12.0)
	Research	27 (1.4)
	Exotics	18 (0.9)
	Equine	10 (0.5)
	Large animal	11 (0.6)
	Retired	16 (0.8)
	Other	91 (4.7)
Position	Full time	1,601 (83.0)
	Part time	329 (17.1)
Role	Associate	1,299 (67.6)
	Owner	622 (32.4)
Gender	Male	447 (23.6)
	Female	1,448 (76.4)
	Transgender/non-binary/other	1 (0.1)
Age (years)	≤ 30	306 (16.1)
	31–40	625 (32.9)
	41–50	416 (21.9)
	51–60	371 (19.5)
	61–70	158 (8.3)
	>70	25 (1.3)
Marital status	Married	1,398 (73.5)
	Single	374 (19.7)
	Divorced	102 (5.4)
	Other	27 (1.4)
Number of children	0	1,199 (62.9)
	1	269 (14.1)
	2	310 (16.3)
	3	103 (5.4)
	4	17 (0.9)
	5	5 (0.3)
	≥6	2 (0.1)

### On-Call

Only 9.8% of respondents (186) had never had on-call duties; slightly more than half [1,008 (58.7%)] currently had on-call duties. The majority of those who reported having on-call duties were female associates (52.55%), followed by female owners (22.02%), male owners (10.91%), and male associates (14.51%). When asked if on-call duties had contributed to leaving a previous job, almost equal numbers said it had been an important factor [234 (33.2%)] or not a factor at all [235 (33.4%)]. One hundred and fifty-four (21.9%) said it had been a moderate factor and [81 (11.5%)] said it had been a minor factor. When asked if lack of on-call duties had ever influenced acceptance of a job, half [356 (51.3%)] reported it as an important factor, 152 (21.9%) reported it had no influence, and the remaining 26.8% reported it as a minimal to moderate factor in their decision. Sixty percent (1,029) indicated that on-call duties would have a major role in their acceptance of a future job, with 25.2% (432) reporting it would play a moderate role, 8.5% (146) a minor role, and 3% (52) no role (3.4% selected “not applicable”).

Of those with on-call duties, a third [321 (32.0%)] had 5–8 nights on-call per month, a fifth [218 (21.8%)] had 1–4, 15.7% (157) had 9–12, 13.8% (138) had 13–16, 3.6% (36) had 17–20, 1.9% (19) had 21–24, and 11.3% (113) had over 24. Half of respondents [505 (50.4%)] reported their on-call shifts typically entailed 1–2 consecutive nights, with 176 (17.6%) being on for 3–4 consecutive nights, 6.0 (60) for 5–6 nights, 15.3 (153) for 7–30 nights, and 10.9% (109) being on for more than 30 nights in a row. On weekends, 60% (604) reported often or always being on-call, with only 1.8% (18) never being on-call over weekends. The impact of being on-call over weekends was rated extremely negative by a third [321 (32.5%)], moderately negative by 59.1% (583), and no impact by 7.2% (71). 1.2% (12) reported it having a positive impact on their weekends. On holidays, just under half [477 (47.3%)] reported sometimes being on-call, with 406 (40.3%) being often or always on-call. The impact reported on holidays was very similar to that of weekends.

Veterinarians on-call were most often contacted directly by clients, with answering services and staff each being the source of calls slightly more than half as often. Calls originating from other veterinarians were less common.

### Compensation

**Figure 1:**
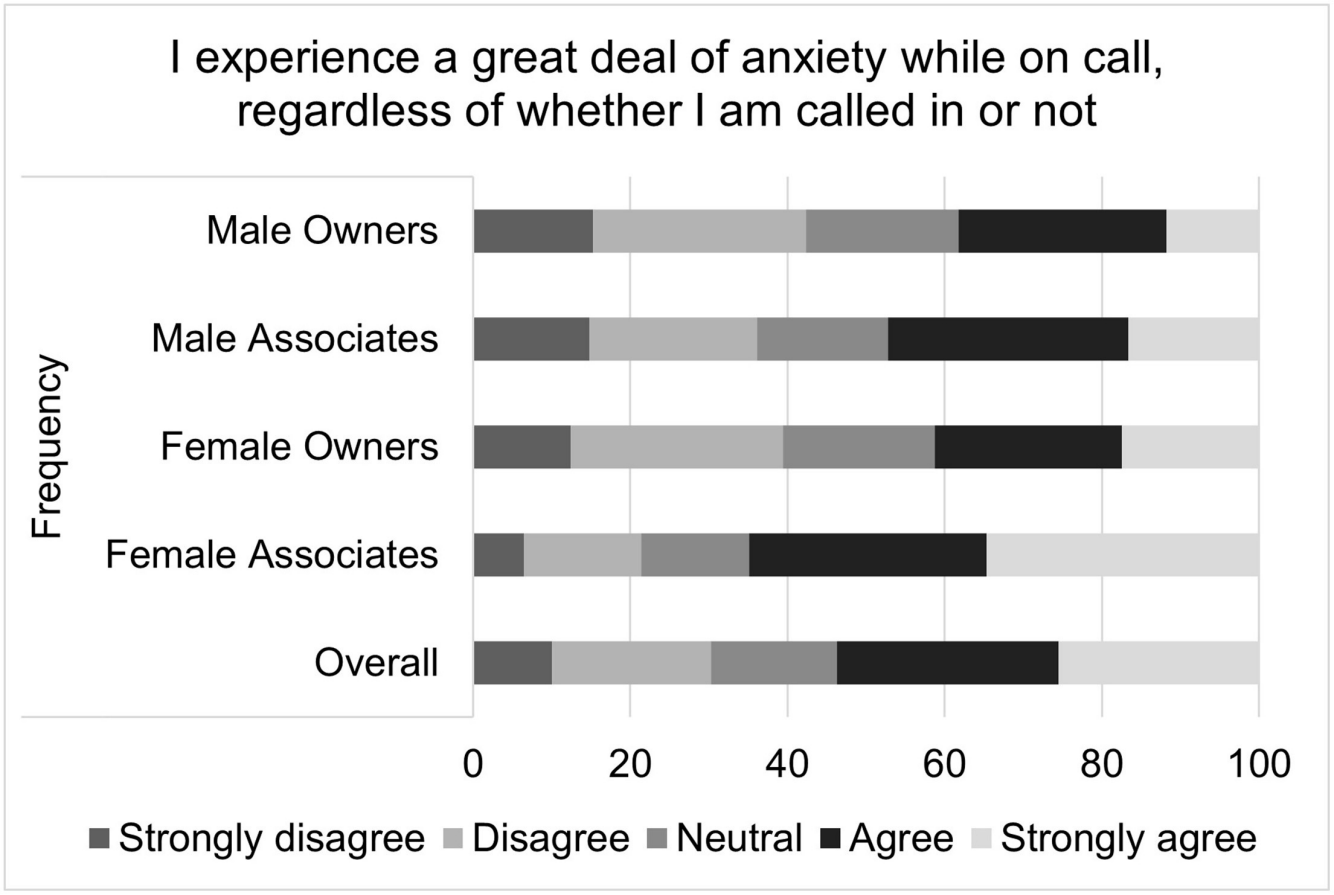
Frequency of having to report to the work site vs. handle matters by phone, during on-call shifts.

The majority [828 (82.14%)] were not compensated merely for being on-call. Slightly more than half of respondents [569 (56.5%)] were compensated only if they were called in. Compensation for being called in was most commonly paid based on the number of cases [246 (43.16%)], followed by the amount billed [200 (35.1%)], “other” [77 (13.5)], and a flat fee [47 (8.3%)]. The relative frequency of being able to handle matters by phone only, vs. having to report to the work site when on-call, is shown in Figure 1. Among respondents who were on-call, 83.8% did not get the next day off if called in the previous night.

### Composite Outcomes

**Figure 2:**
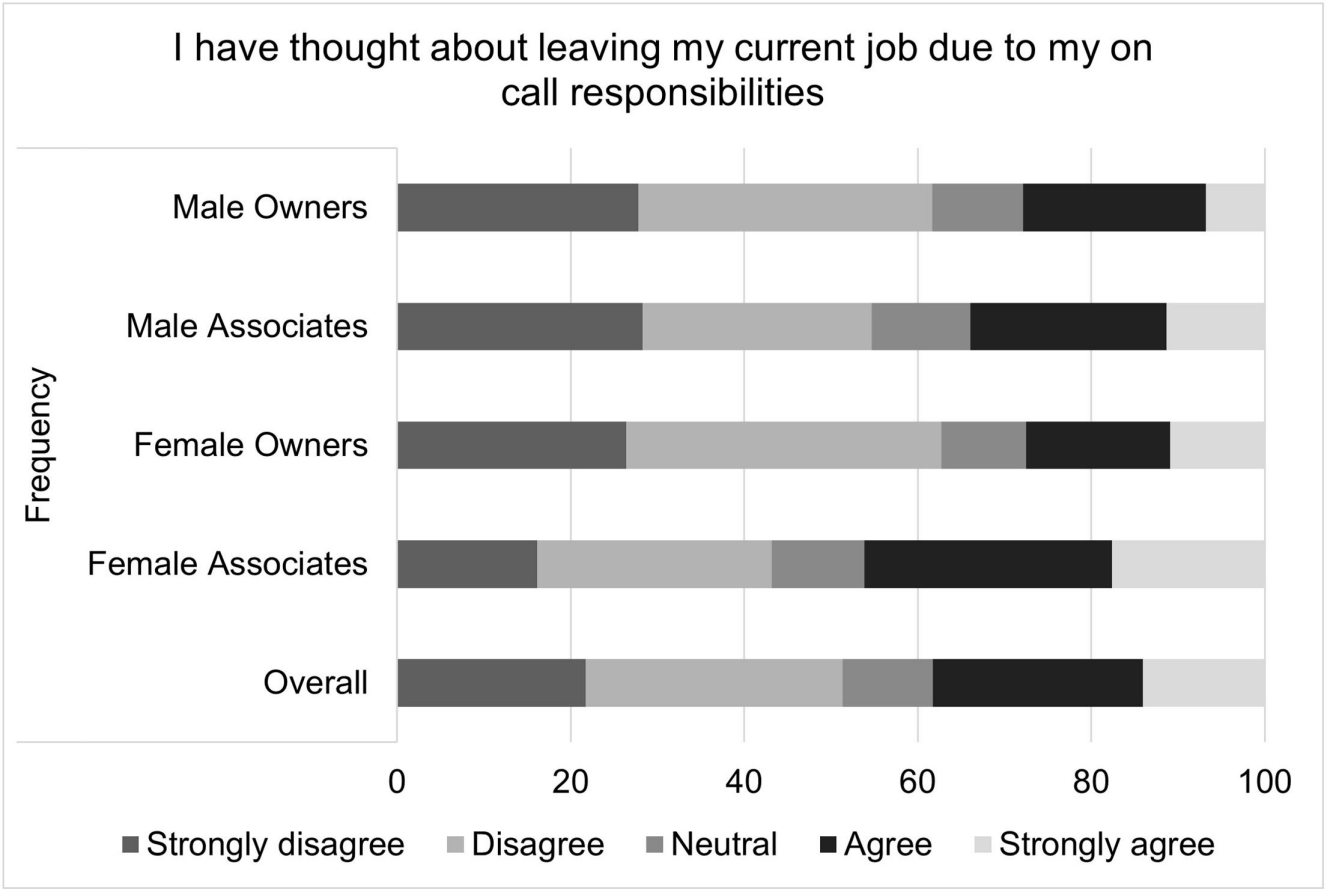
Impact of on-call duties on job satisfaction, as reported by male and female practice owners and associate veterinarians.

**Figure 3:**
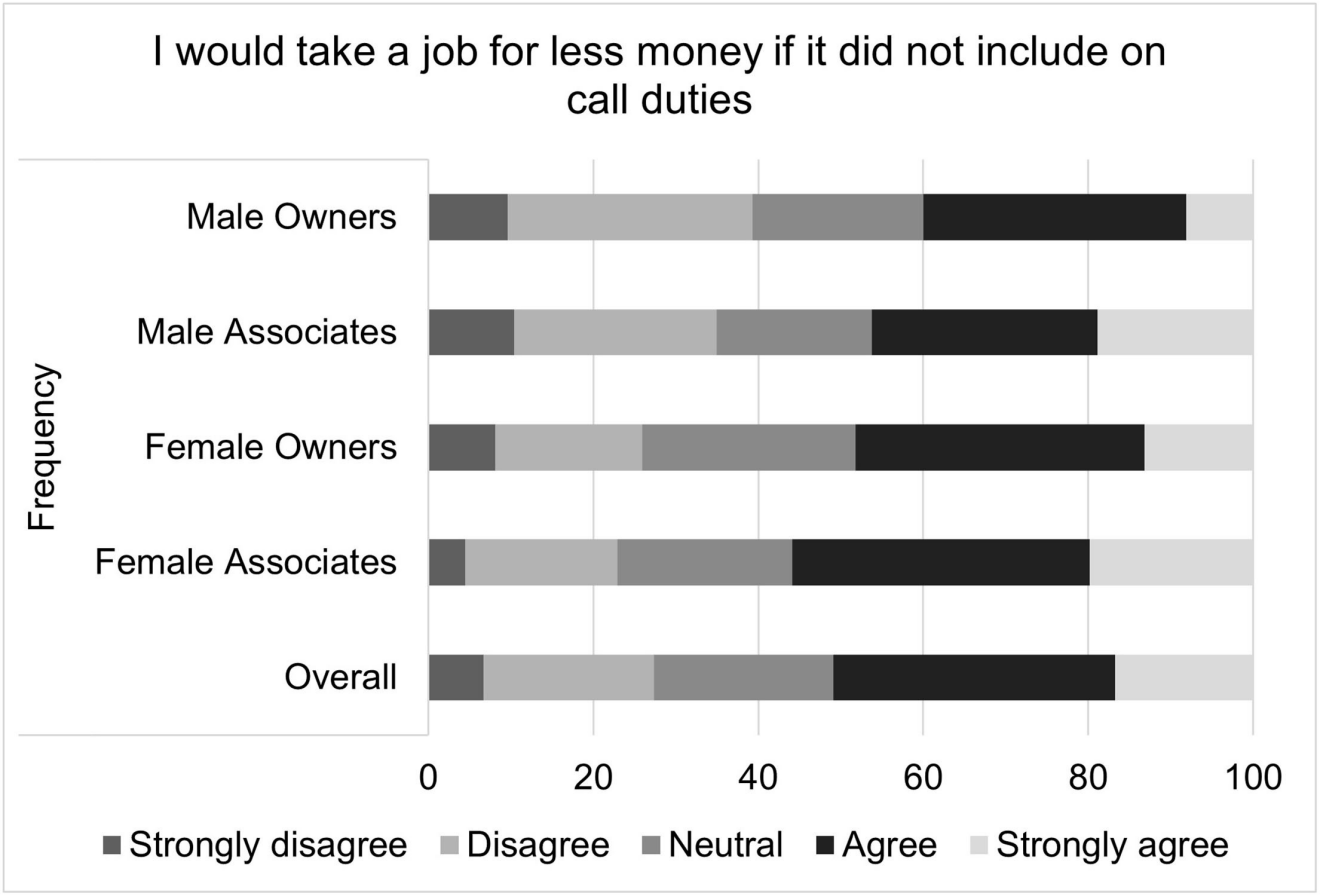
Willingness to accept a lower paying job in lieu of on-call duties, as reported by male and female practice owners and associate veterinarians.

**Figure 4:**
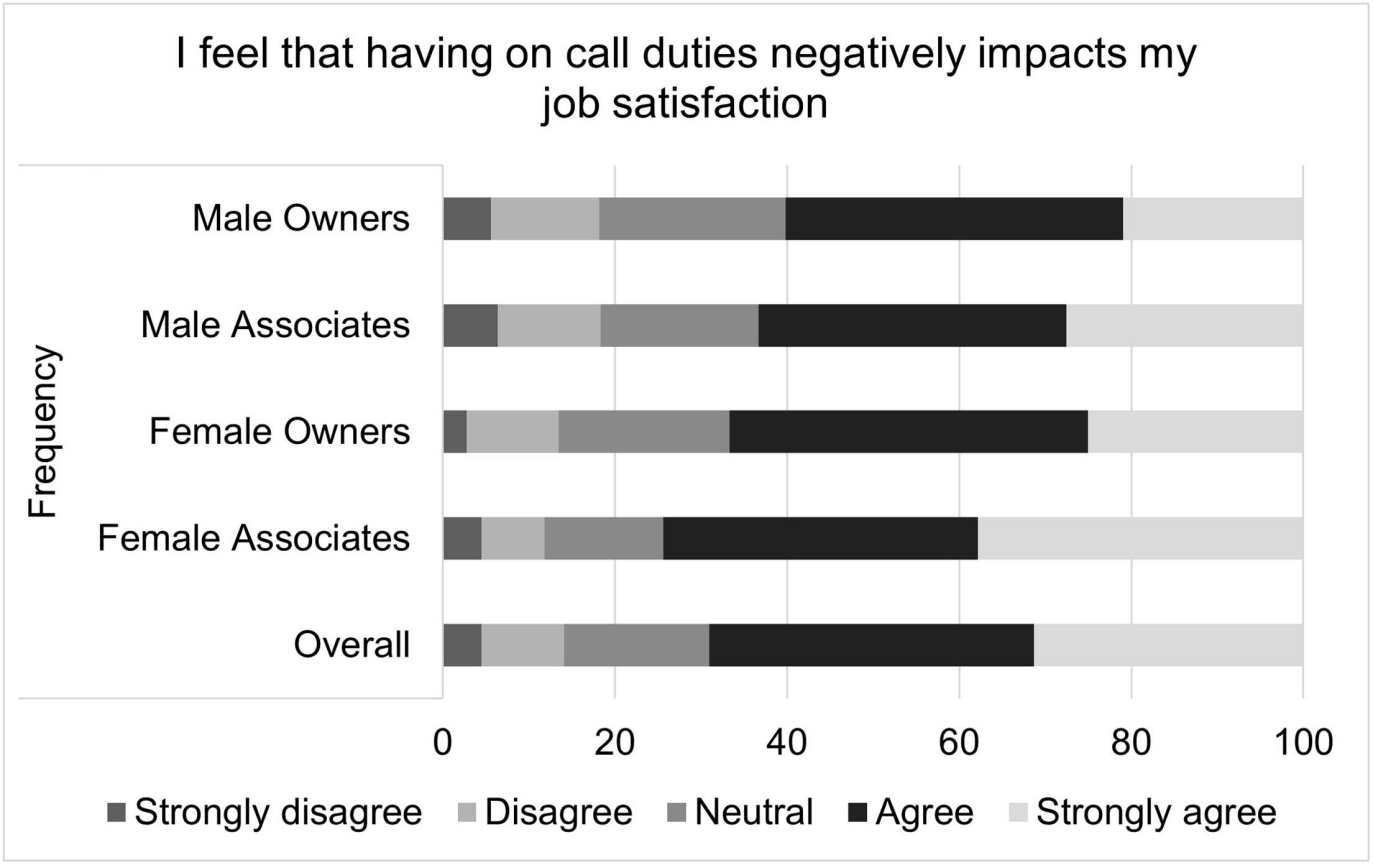
Willingness to leave current job due to on-call responsibilities, as reported by male and female practice owners and associate veterinarians.

For the composite score of the impact on-call shifts on perceived job satisfaction, the median score was 4 with an interquartile range of 10 and a total range of 31. Higher job satisfaction scores correspond to greater job satisfaction. Multiple linear regression found that gender (*p* = 0.0311), associate status (*p* <0.0001), and age (*p* = 0.0293) were all significantly associated with on-call related job satisfaction (Table 2). Females, compared to males, had on-call related job satisfaction that was on average 1.27 points lower than that of males. Associates, compared to owners, had an on-call related job satisfaction score that was an average of 3.05 points lower. For each decade increase in age, job satisfaction increased by 0.51 points. Visual inspection of the residual QQ plot and residual x predicted plot indicated that the assumptions of normality and homoscedasticity were adequately met. No concerns about collinearity were appreciated. Specific questions that comprise the on-call shifts related job satisfaction score are illustrated in Figures 2–4.

**Table 2:** Exploratory factor analysis.

**Outcome**	**Covariate**	**Parameter estimate**	**95% CI**	***P*-value**
**Job satisfaction**				
	Gender	−1.27	(−2.42, −0.12)	0.0311
	Associate status	−3.05	(−4.18, −1.92)	<0.0001
	Age (decadal)	0.51	(0.05, 0.96)	0.0293
**Well-being**				
	Gender	1.14	(0.37, 1.92)	0.0039
	Associate status	1.08	(0.31, 1.84)	0.0057
	Age (decadal)	−0.74	(−1.05, −0.44)	<0.0001
**Relationships**				
	Gender	0.12	(−0.17, 0.42)	0.4115
	Associate status	0.36	(0.06, 0.65)	0.0173
	Age (decadal)	−0.15	(−0.27, −0.03)	0.0159

*Multiple linear regression*.

**Figure 5:**
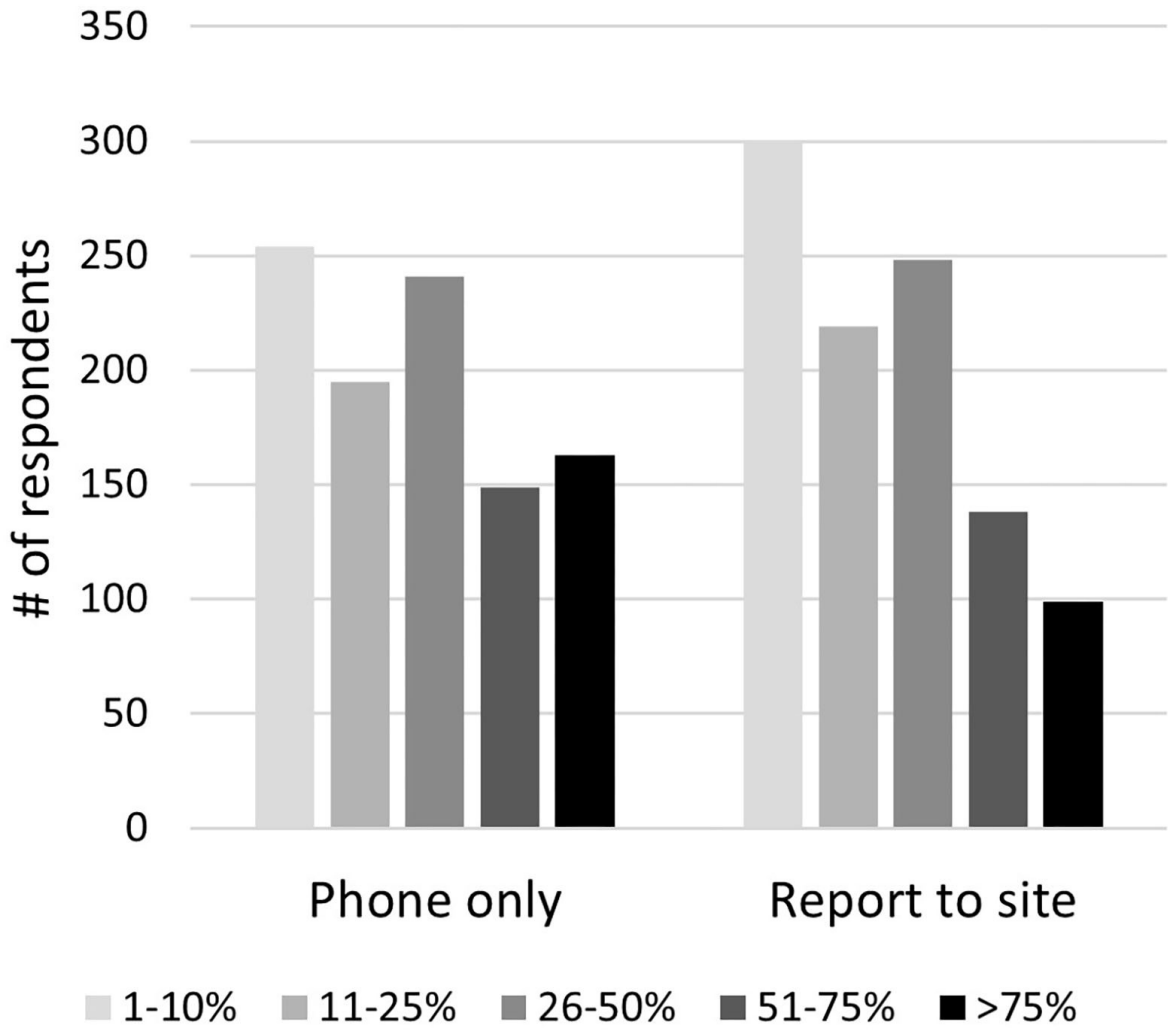
Anxiety levels experiences while on-call, as reported by male and female practice owners and associate veterinarians.

**Figure 6:**
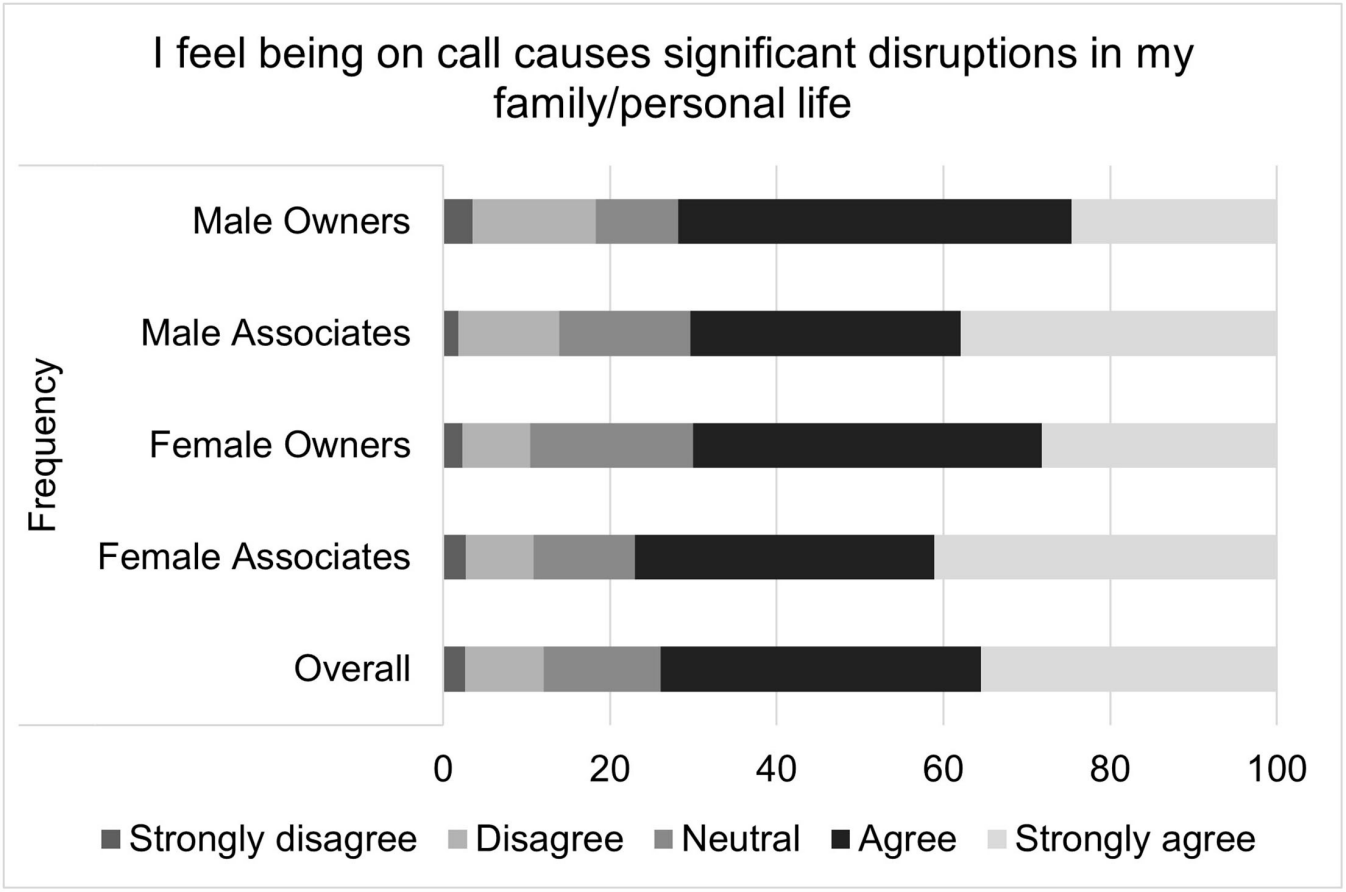
Perceived negative effects of on-call duties on physical health, as reported by male and female practice owners and associate veterinarians.

**Figure 7:**
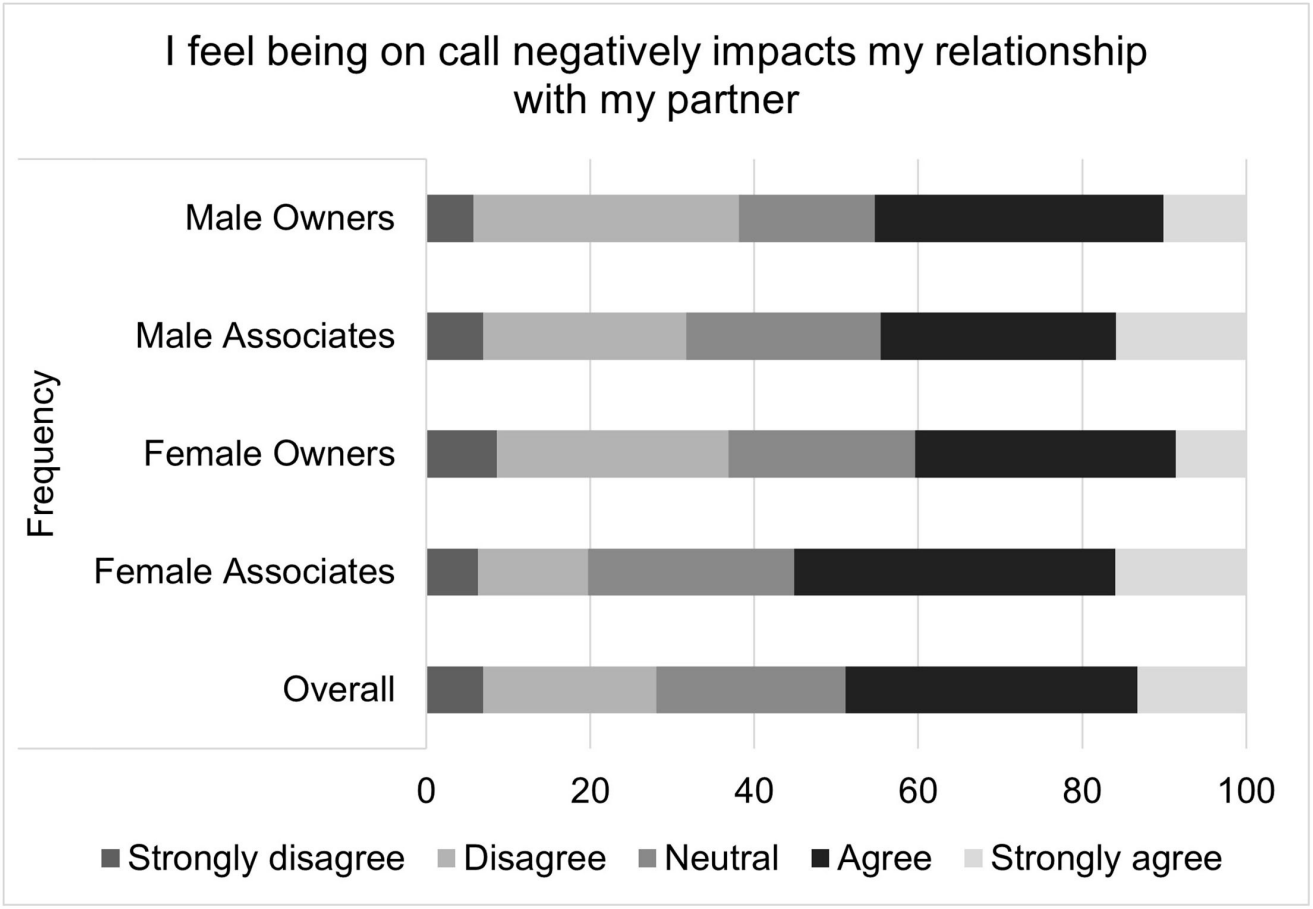
Perceived negative effects of on-call duties on psychological health, as reported by male and female practice owners and associate veterinarians.

**Figure 8:**
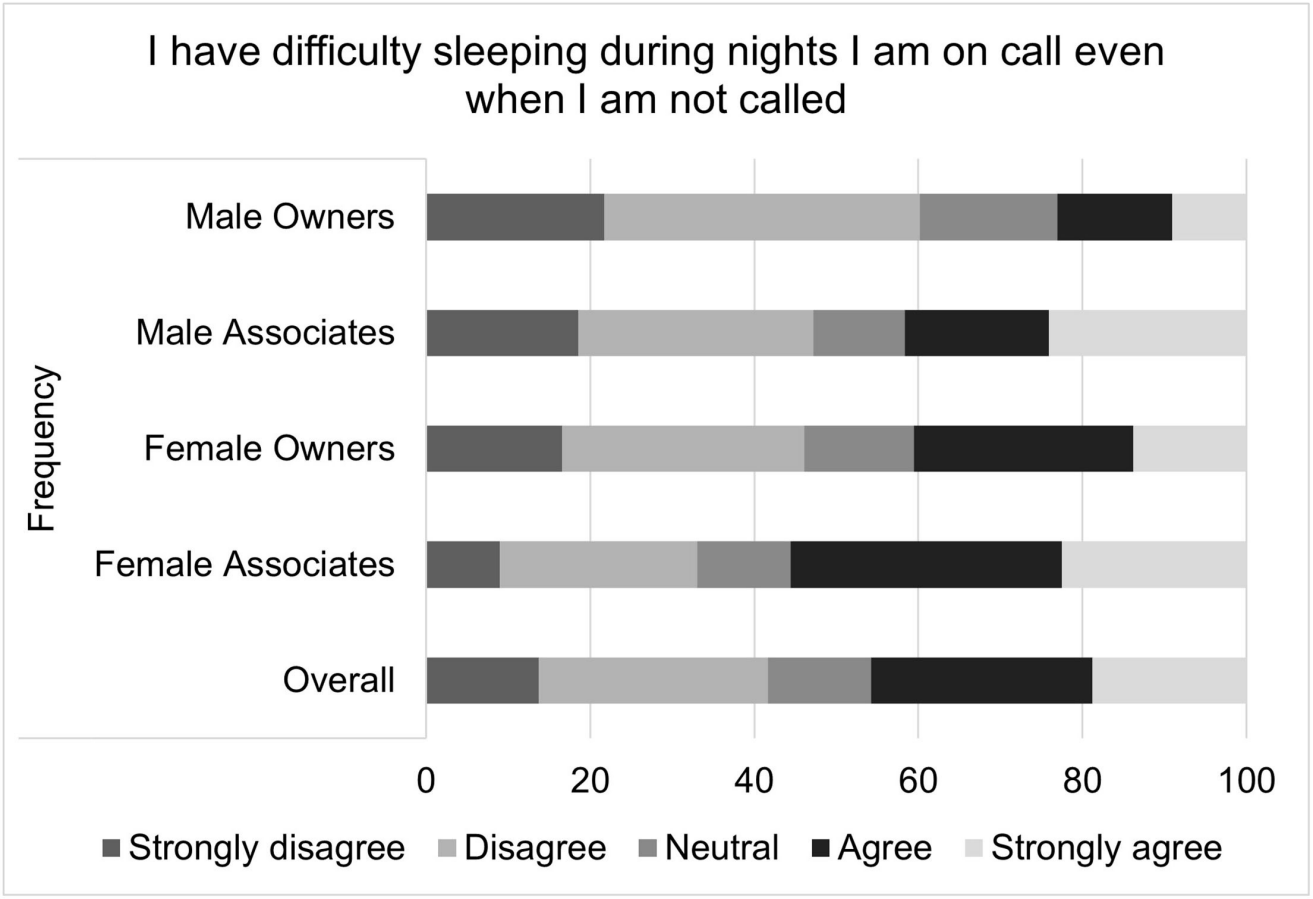
Perceived inability to relax while on-call, as reported by male and female practice owners and associate veterinarians.

**Figure 9:**
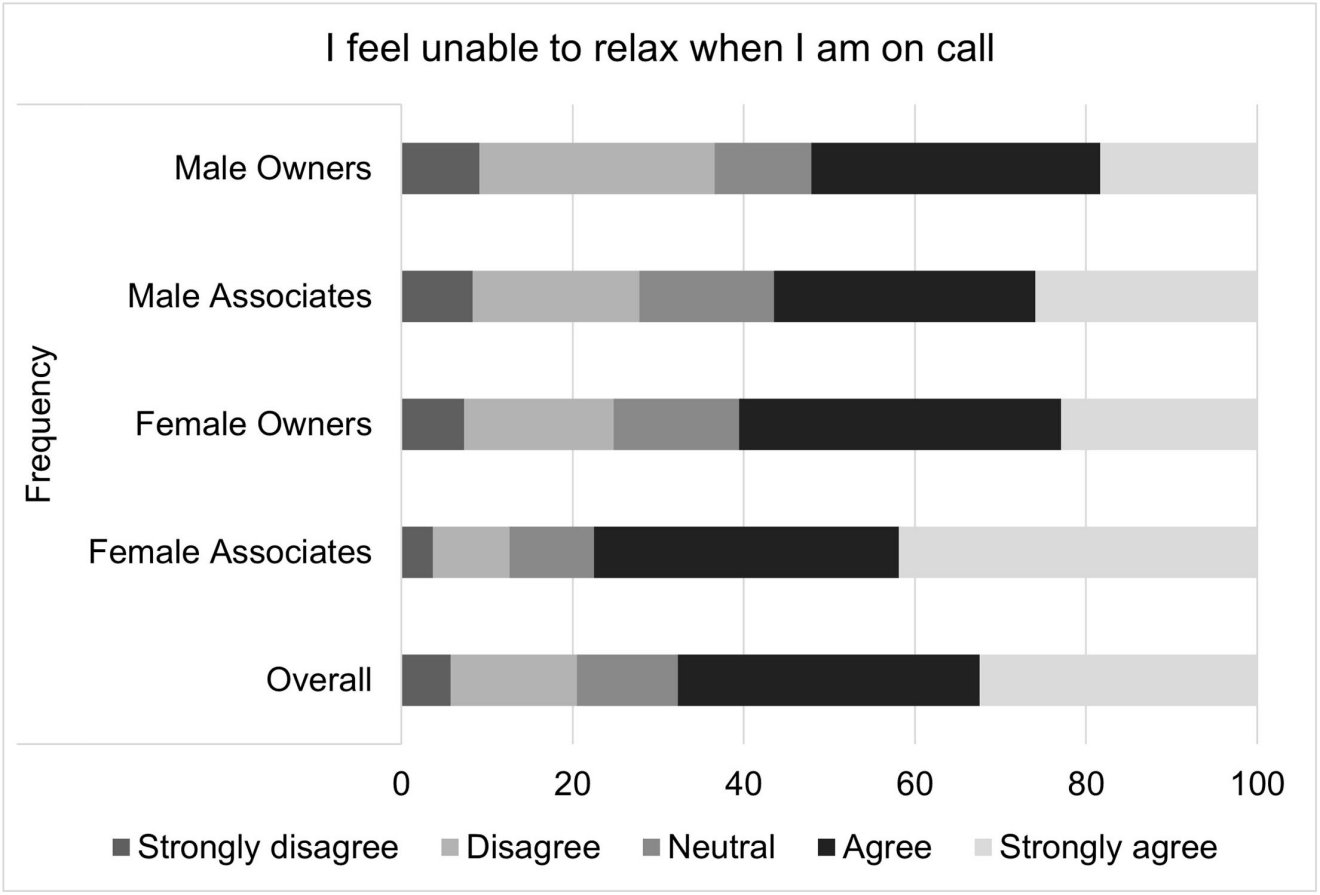
Sleep difficulties while on-call, as reported by male and female practice owners and associate veterinarians.

For on-call related well-being scores, the median score was 18 with an interquartile range of 8 and a total range of 20. It is important to note that higher well-being scores indicate poorer health. The multiple linear regression residual QQ plot and residual × predicted plot suggested that non-normality and heteroscedasticity might be important. Transformation did not improve the normality of residuals, so bootstrapping with robust regression was performed. The standard error estimates produced by this method did not differ substantially from those of the standard multiple linear regression, so the standard regression results are reported. Again, gender (*p* = 0.0039), associate status (*p* < 0.0057), and age (*p* < 0.0001) were all significantly associated with the outcome. Specifically, females on average had an on-call related well-being score that was 1.15 points higher than that of males; associates on average had a score that was 1.08 points higher than owners; and the score decreased by 0.745 points for each additional decade of age. Specific questions that comprise the on-call shifts related job satisfaction score are illustrated in Figures 5–9.

**Figure 10:**
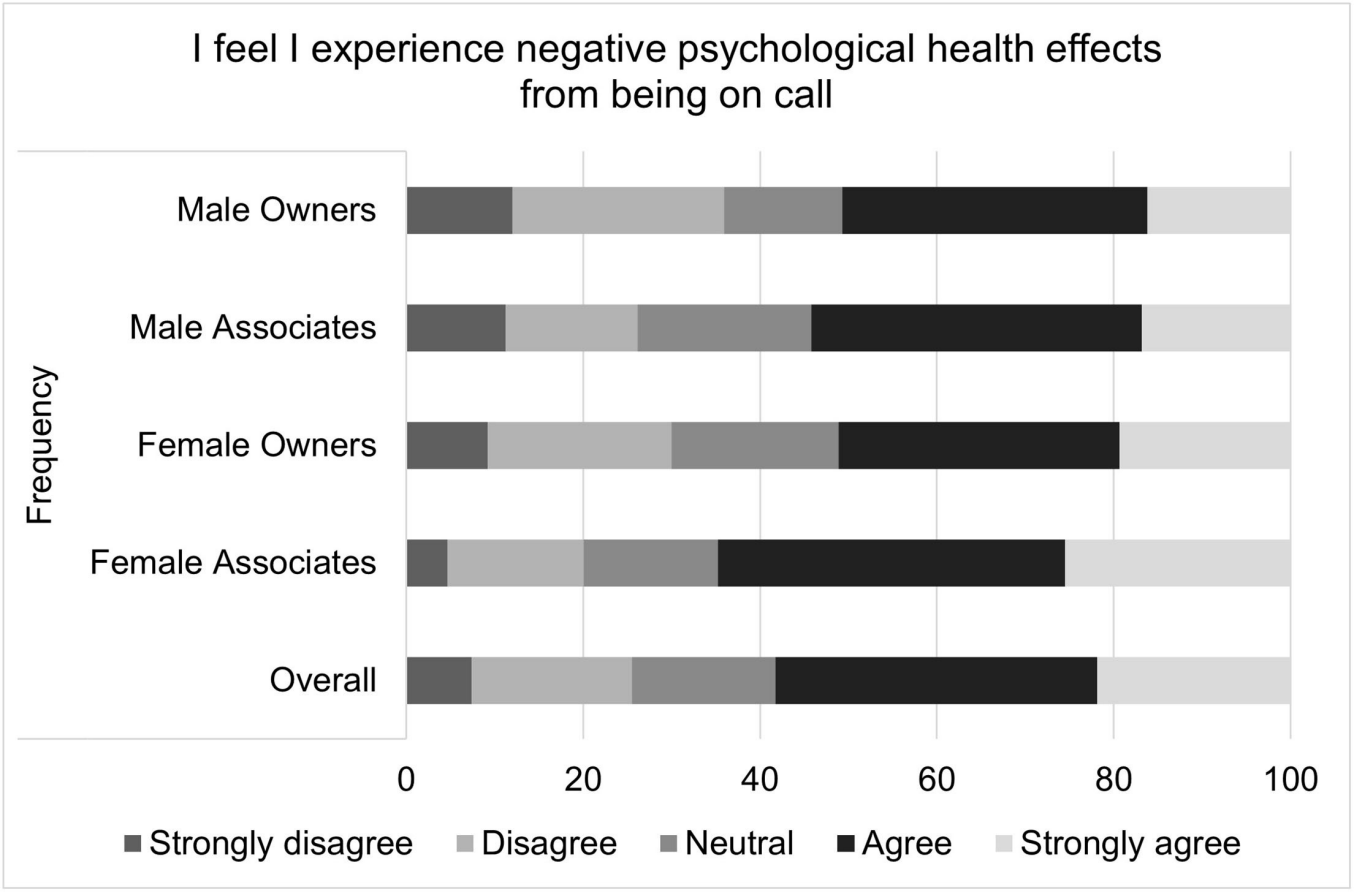
Extent to which being on-call negatively impacts personal relationships, as reported by male and female practice owners and associate veterinarians.

**Figure 11:**
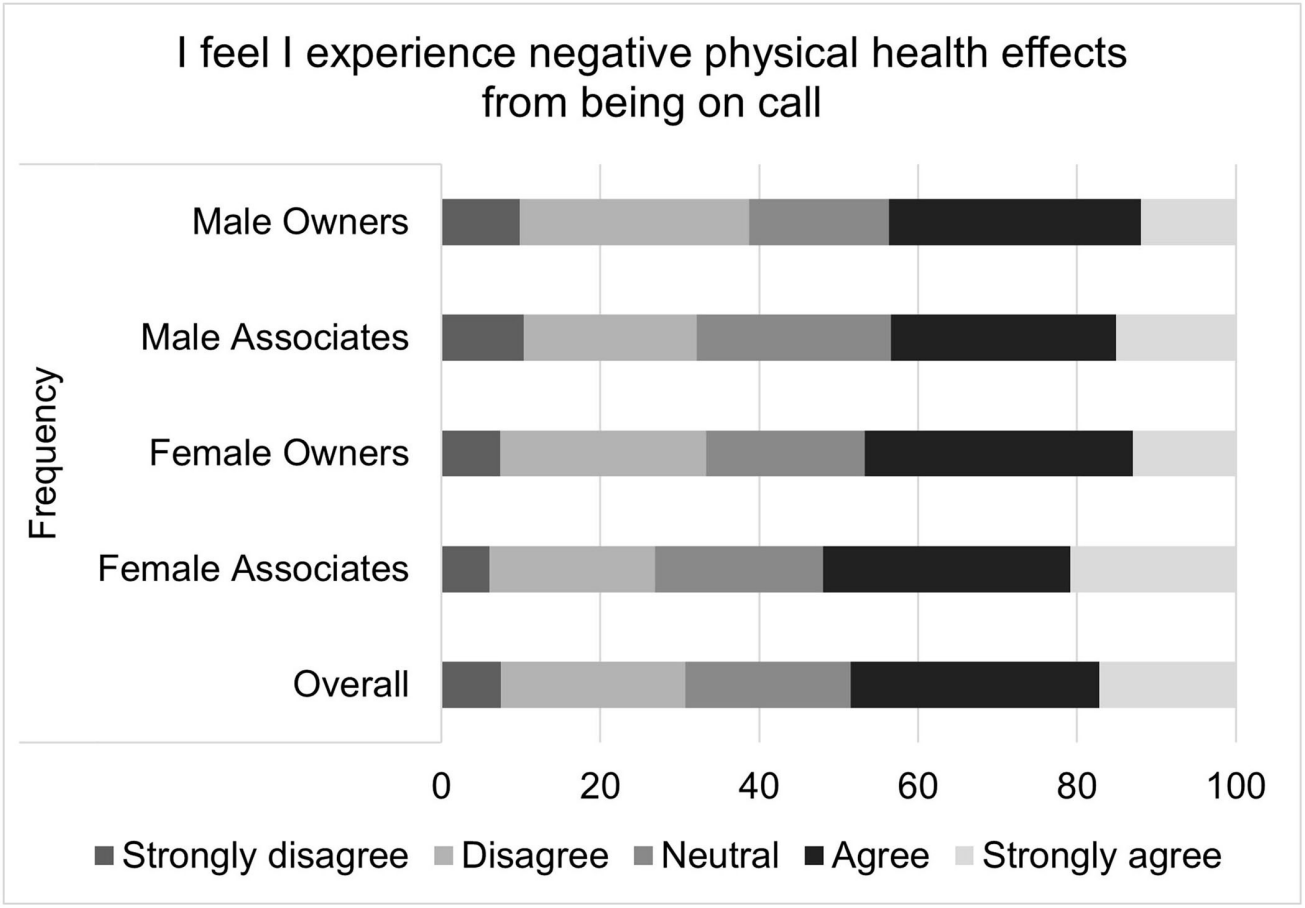
Extent to which being on-call causes significant disruptions in family/personal life, as reported by male and female practice owners and associate veterinarians.

For the on-call related relationship composite, the mean score was 7.21 ± 1.91. When assessing only those who indicated they were in a relationship, the mean score was 7.13 ± 1.94. It is important to note that higher relationship scores indicate poorer relationship health. Further analysis was conducted on those who indicated they were in a relationship. The multiple linear regression residual QQ plot and residual × predicted plot indicated acceptable approximations of normality and homoscedasticity, particularly given the large size of the data set (central limit theorem). Associate vs. owner status was a borderline significant predictor (*p* = 0.0715), and age (*p* = 0.0011) was a significant predictor, although gender was not (*p* = 0.8143). Associates had an on-call related relationship score that was on average 0.29 points higher than owners, while each additional decade decreased the relationship score by 0.23 points. Specific questions that comprise the on-call shifts related job satisfaction score are illustrated in Figures 10, 11.

### Exploratory Factor Analysis and Instrument Internal Consistency

Exploratory factor analysis found that one factor was insufficient (*p* < 0.0001); visual inspection of the scree plot and Eigenvalues suggested either two or three latent factors. Utilizing three factors resulted in several survey questions with substantial loading onto multiple factors, whereas two factors minimized this issue and were found to be sufficient (Table 3; *p* < 0.0001). The two latent factors clearly segregate based on perception of on-call, with one assessing negative aspects of being on call (e.g., being on call increases anxiety) and the other assessing positive aspects (e.g., enjoyment of the types of cases seen when on call). The question querying whether adequate assistance was available while on call did not load adequately on either factor, perhaps because this is a slightly more objective item.

**Table 3:** Regression analyses.

	**Factor loading**	
**Likert variable**	**Factor 1—Negative aspects of on-call work**	**Factor 2—Postive aspects of on-call work**
Negative psychological effects	0.80552	−0.31594
Negative health effects	0.73877	−0.22891
Causes anxiety	0.67987	−0.35558
Cannot relax	0.66498	−0.33187
Causes insomnia	0.65727	−0.21722
Disrupts life	0.64283	−0.27125
Negatively affects relationship	0.63256	−0.18552
Have thought about leaving job due to on-call	0.57816	−0.40762
Would take less pay for no on-call duty	0.51561	−0.42017
Negatively impacts job satisfaction	0.48825	−0.49541
Enjoy being on call	−0.23185	0.77083
Enjoy on-call cases	−0.18122	0.71137
Just part of the job	−0.35051	0.57798
Important to be on call for my patients	−0.18715	0.57122
Extra pay makes it worth it	−0.36502	0.49292
Adequate assistance available	−0.19759	0.24315

*Selection method = maximum likelihood, rotation method = varimax, variance explained (negative aspects) = 93.9%, variance explained (positive aspects) = 68.6%, Tucker and Lewis's reliability coefficient = 0.867*.

Internal consistency of the survey instrument was assessed via Cronbach's alpha, which was only moderate for the overall survey at 0.562 (standardized 0.544). However, when separated into two groups based on exploratory factor analysis, Cronbach's alpha was acceptable at 0.913 (0.913) for negative aspects and 0.768 (0.777) for positive aspects. Cronbach's alpha for all surveys with answers for at least 80% of Likert items, with missing data addressed via the mean replacement method, was 0.588 (0.566) overall, 0.906 (0.906) for negative items, and 0.748 (0.756) for positive items.

## Discussion

This study is the first to explore the topic of on-call shifts on veterinarians' job satisfaction, well-being and personal relationships. Nearly all participants had on-call duties at some point in their career and over half currently assumed on-call duties. The majority of those who reported on-call duties were female associates. Nearly a third of respondents had 5–8 on-call shifts per month and most reported having 1–2 consecutive nights. Only 18% of participants reported receiving a stipend for simply being on-call, in the absence of performing clinical duties. The vast majority of participants were only compensated for on-call time when they had to report for work. Compensation was most commonly based on number of cases seen followed by the amount billed. Regardless of pay structure, over 75% of respondents reporting feeling the money was not worth the inconvenience. Perhaps this is in part due to the fact that most surveyed veterinarians felt that being on-call significantly altered their recreational plans, and negatively impacted both their weekends and their holidays.

Composite scores were created to assess the impact of on-call shifts on job satisfaction, well-being and personal relationships. On-call shifts was reported by many to have a negative impact on job satisfaction. This was especially pronounced for female associates. For example, over half of all participants reported agreeing or strongly agreeing that on-call duties negatively impacted their job satisfaction, but female associates were most likely to endorse this sentiment, followed by female owners, male associates and then male owners. Female associates were also more likely to agree with the statement “I have thought about leaving my current job due to my on-call responsibilities” as well as the statement “I would take a job for less money if it did not include on-call duties.”

It has been suggested that being on-call, regardless of whether one is actually called in, has a negative impact on the recovery needed to maintain mental and physical health. This is due to the inability to cognitively detach from work, and a continued psychophysical activation state (48). Yet, in most countries, off-site on-call duty is considered rest time. Only when an employee is called to work are they viewed as working (37). Yet, being on-call impacts rest time in several ways: it restricts what employees can do during their leisure time, places geographical limits on their location (e.g., having to stay within a certain radius from the workplace) and necessitates the restriction of some behaviors (e.g., drinking alcohol). Control, in this context, refers to the degree to which a person can select their leisure activities (49), but, being on-call impacts this sense of control. Iso-Ahola (50) noted that the ability to participate in intrinsically desirable activities is one of the basic features of leisure time. Greater perceived control over leisure time has been associated with greater well-being, but an absence of control has been found to relate to a higher level of stress (16, 49, 51–53). Psychological detachment entails being able to mentally disengage from work and is critical to help prevent fatigue, burnout, and emotional exhaustion (37, 54, 55). Yet, being on-call has a negative impact on recovery (1, 4, 16, 37).

The impact of on-call restrictions was reflected in the proportion of female associates who endorsed statements included in the well-being composite score. These questions include “I have difficulty sleeping during nights I am on-call even when I am not called,” “I feel unable to relax when I am on-call,” “I feel I experience negative psychological health effects from being on-call,” “I feel I experience negative physical health effects from being on-call,” and “I experience a great deal of anxiety while on-call, regardless of whether I am called in.”

This study's results associating increased anxiety and sleep disturbances to on-call shifts are supported by previous research. Sprajcer (56), for example, found that pre-bed anxiety was significantly higher on nights participants were on-call compared with nights they were not on-call. Numerous researchers have drawn attention to the negative impact of sleep deprivation on health care providers as well as their patients (2, 29). Medical professionals are required to exhibit consistently high levels of psychomotor performance and cognitive function yet sleep deprivation has been found to significantly impact these areas, leading to concerns about health care providers' performance and the resultant impact on patient safety (29, 57–59). Wilhelm (60) found that sleep deprivation after even just one on-call night can lead to a significant reduction in alertness and an increase in resultant hazards. Kaneita's study of Japanese physicians (19) supported these results, demonstrating the association between on-call shifts and excessive daytime sleepiness. The sleep deprivation from on-call shifts can take a physical and psychological toll. Being on-call has been associated with lower mood (17) and increased stress and distress (13, 61, 62).

Another critical area to heed is performance level when veterinarians are called into work. The fact that most on-call work is an abrupt switch from a non-work environment (including sleep) to a high demand, emergency situation, creates the possibility of less satisfactory performance than that demonstrated on regular shifts. As Ziebertz (37) noted, this abrupt change can make it challenging to perform at top levels. Lockley (23), for example, found that on-call medical residents made 36% more serious medical errors and 300% times more fatigue related medical errors that led to patient death when compared to those working 16 h shifts. Another study by Williams (21) found similar results when examining anesthesia residents. Although performance was not assessed in the current study, the relationship between sleep deprivation, performance, and error rates is an important aspect of on-call shifts.

The last composite scale, comprised of two relationship oriented questions, reflected similar trends to the two previous composite scales, even though the overall on-call relationship scale scores did not significantly differ between men and women. Female associates, however, agreed more often than female owners or male owners or associates when asked “I feel being on-call negatively impacts my relationship with my partner” and “I feel being on-call causes significant disruptions in my family/personal life.”

The impact that on-call shifts has on personal lives and relationships has been noted in several previous studies (2, 8, 63). Papp found that sleep deprivation had a major impact on health professionals' personal life while other studies have shown that being on-call can negatively impact intimate relationships and family connections (38, 39, 63, 64). Perhaps part of this impact might be due to the fact that the demands of being on-call can result in less time available for household activities and social activities (4). A lack of time to do household activities can strain relationships and add additional stress. This might be especially true for females since they do the majority of housework (65).

Taken together, these results suggest that the majority of veterinarians surveyed feel that being on-call is detrimental in terms of job satisfaction, well-being and personal relationships. Given the fact that most clinical veterinarians are expected to work on-call shifts, and yet, feel it negatively impacts numerous facets in their lives, suggests that this is an important area to more closely examine.

One area that should perhaps be reexamined is the definition of off-site on-call shifts as leisure time. It is suggested that, due to its impact on recovery, time spent being on-call is not actually rest time. As the effort–recovery model (41) suggests, adequate recovery is critical for employees' mental and physical health (66). Burnout, the consequence of prolonged occupational stress resulting in emotional exhaustion and a reduced sense of personal accomplishment can negatively impact the quality of care provided (67) and personal well-being (68). Inadequate recovery increases the chances of burnout, a significant issue within veterinary medicine (69, 70). It has been suggested that the field as a whole give increased attention to veterinarians' well-being and allocate resources toward support efforts aimed at helping veterinarians address occupational stress, compassion fatigue and burnout (71–74). This is especially important as the demographics of veterinarians are undergoing significant changes.

It is suggested that young veterinarians choose their career paths based on numerous factors including visions of their future lifestyle. Given the fact that many participants in this study noted that on-call duties have played a role in their decisions about previous and future jobs, it is clear that many veterinarians are examining how on-call demands impact their visions of a future career and work/life balance. The proportion of physicians willing to take on-call duty is falling (75), likely due to these same considerations. Many young physicians view a work/life balance as critically important and are making career decisions to support this lifestyle (75, 76). The results of the current study support the premise that veterinarians are making similar assessments. Veterinary medicine is becoming a predominately female occupation and these considerations are perhaps even more pronounced, as supported by the results indicating that women are impacted more from on-call shifts than men in the areas of well-being and job satisfaction.

Supporting veterinarians is critically important for the future of the profession. Current research has validated what many have known; that veterinary clinical positions are highly stressful (71, 72, 77–79) and this stress can lead to psychological distress and increased suicide risk (72, 73). Both male and female veterinarians have higher suicide rates than the general population and for women in clinical practice, the difference is even greater. Female veterinarians in clinical practice are five times as likely to die by suicide as the general population (71). Given that over 60% of US veterinarians and over 80% of US veterinary students are female, the fact that on-call work appears to impact women more than men (80) is a critical discovery and one that necessitates a hard look at current practices.

As with any research, our study has limitations. Even though the sample size was adequate, it was only a small percentage of total VIN members, which might bias the results. It is possible that clinicians who feel strongly about on-call shifts might have been more likely to respond. Yet, our sample appears to be representative of clinical veterinarians. The measures used to assess the impact of on-call shifts on job satisfaction and personal relationship were created for this study. Validating these instruments in the future will be important next steps in researching this topic. Additionally, the survey asked for some personal mental health information that some participants may have not wanted to share. Lastly, it is important to note that this survey marks one moment in time and we suggest caution about generalizing to other time periods. Future research, both qualitative and quantitative, is needed to further develop our understanding of the impact of on-call shifts.

## Conclusions

Results of this study suggest that at least part of this support should be exploring options designed to make on-call shifts more manageable. Suggestions include family and household support, childcare options, and flexible scheduling. Bamberg et al. (4), for example, suggested shorter periods of availability for fewer employees who are contacted more frequently as one option to minimize on-call stress. Some veterinary hospitals might want to explore hiring temporary or relief staff to help with on-call shifts, or next day shifts after a veterinarian has been called in to work. Exploring ways to mitigate the stress of on-call shifts is necessary to ensure the mental and physical health of veterinarians as well as the safety and medical care of their patients. What appears clear is that for on-call work, the rationale that ‘we've always done it this way' is no longer acceptable.

## Data Availability Statement

The raw data supporting the conclusions of this article will be made available by the authors, without undue reservation.

## Ethics Statement

The studies involving human participants were reviewed and approved by Colorado State University Institutional Review Board. Written informed consent for participation was not required for this study in accordance with the national legislation and the institutional requirements.

## Author Contributions

All authors listed have made a substantial, direct and intellectual contribution to the work, and approved it for publication.

## Conflict of Interest

The authors declare that the research was conducted in the absence of any commercial or financial relationships that could be construed as a potential conflict of interest.

## Publisher's Note

All claims expressed in this article are solely those of the authors and do not necessarily represent those of their affiliated organizations, or those of the publisher, the editors and the reviewers. Any product that may be evaluated in this article, or claim that may be made by its manufacturer, is not guaranteed or endorsed by the publisher.

## References

[1] Ziebertz CM Beckers DGJ Van Hooff MLM Kompier MAJ Geurts SAE. The effect on sleep of being on-call: an experimental field study. J Sleep Res. (2017) 26:809–15. 10.1111/jsr.12519 28349565

[2] Hall SJ Ferguson SA Turner AI Robertson SJ Vincent GE Aisbett B. The effect of working on-call on stress physiology and sleep: a systematic review. Sleep Med Rev. (2017) 33:79–87. 10.1016/j.smrv.2016.06.001 27426960

[3] Ferguson SA Paterson JL Hall SJ Jay SM Aisbett B. On-call work: to sleep or not to sleep? It depends. Chronobiol Int. (2016) 33:678–84. 10.3109/07420528.2016.1167714 27070367

[4] Bamberg E Dettmers J Funck H Krähe B Vahle-Hinz T. Effects of on-call work on well-being: results of a daily survey 1. Appl Psychol Heal Well Being. (2012) 4:299–320. 10.1111/j.1758-0854.2012.01075.x 23081765

[5] Nicol A-M Botterill JS. On-call work and health: a review. Environ Heal. (2004) 3:15. 10.1186/1476-069X-3-15 15588276 PMC539298

[6] Sun M Feng W Wang F Li P Li Z Li M. Meta-analysis on shift work and risks of specific obesity types. Obes Rev. (2018) 19:28–40. 10.1111/obr.12621 28975706

[7] Ferri P Guadi M Marcheselli L Balduzzi S Magnani D Di Lorenzo R. The impact of shift work on the psychological and physical health of nurses in a general hospital: a comparison between rotating night shifts and day shifts. Risk Manag Healthc Policy. (2016) 9:203–11. 10.2147/RMHP.S115326 27695372 PMC5028173

[8] Lindfors PM Nurmi KE Meretoja OA Luukkonen RA Viljanen A-M Leino TJ. On-call stress among Finnish anaesthetists. Anaesthesia. (2006) 61:856–66. 10.1111/j.1365-2044.2006.04749.x 16922752

[9] Heponiemi T Puttonen S Elovainio M. On-call work and physicians' well-being: testing the potential mediators. Occup Med. (2014) 64:352–7. 10.1093/occmed/kqu036 24659108

[10] Dall'Ora C Griffiths P Ball J Simon M Aiken LH. Association of 12 h shifts and nurses' job satisfaction, burnout and intention to leave: findings from a cross-sectional study of 12 European countries. BMJ Open. (2015) 5:e008331. 10.1136/bmjopen-2015-008331 26359284 PMC4577950

[11] Maruyama S Morimoto K. Effects of long workhours on life-style, stress and quality of life among intermediate Japanese managers. Scand J Work Environ Health. (1996) 22:353–9. 8923608 10.5271/sjweh.153

[12] Van Ham I Verhoeven AAH Groenier KH Groothoff JW De Haan J. Job satisfaction among general practitioners: a systematic literature review. Eur J Gen Pract. (2006) 12:174–80. 10.1080/13814780600994376 17127604

[13] Appleton K House A Dowell A. A survey of job satisfaction, sources of stress and psychological symptoms among general practitioners in Leeds. Br J Gen Pract J R Coll Gen Pract. (1998) 48:1059–63. 9624747 PMC1410027

[14] Virtanen M Jokela M Nyberg ST Madsen IEH Lallikka T Ahola K. Long working hours and alcohol use: systematic review and meta-analysis of published studies and unpublished individual participant data. BMJ. (2015) 350:g7772. 10.1136/bmj.g7772 25587065 PMC4293546

[15] Chambers R Belcher J. Predicting mental health problems in general practitioners. Occup Med. (1994) 44:212–6. 7949065 10.1093/occmed/44.4.212

[16] Dettmers J Vahle-Hinz T Bamberg E Friedrich N Keller M. Extended work availability and its relation with start-of-day mood and cortisol. J Occup Health Psychol. (2016) 21:105–18. 10.1037/a0039602 26236956

[17] Rankin HJ Serieys NM Elliott-Binns CP. Determinants of mood in general practitioners. Br Med J. (1987) 294:618–20. 3103834 10.1136/bmj.294.6572.618 PMC1245659

[18] Kuhn G. Circadian rhythm, shift work, and emergency medicine. Ann Emerg Med. (2001) 37:88–98. 10.1067/mem.2001.111571 11145778

[19] Kaneita Y Ohida T. Association of current work and sleep situations with excessive daytime sleepiness and medical incidents among Japanese physicians. J Clin sleep Med JCSM Off Publ Am Acad Sleep Med. (2011) 7:512–22. 10.5664/JCSM.1322 22003348 PMC3190852

[20] Gaba DM Howard SK. Fatigue among Clinicians and the Safety of Patients. N Engl J Med. (2002) 347:1249–55. 10.1056/NEJMsa020846 12393823

[21] Williams GW Shankar B Klier EM Chuang AZ El Marjiya-Villarreal S Nwokolo OO. Sensorimotor and executive function slowing in anesthesiology residents after overnight shifts. J Clin Anesth. (2017) 40:110–6. 10.1016/j.jclinane.2017.04.002 28625430

[22] Estabrooks CA Cummings GG Olivo SA Squires JE Giblin C Simpson N. Effects of shift length on quality of patient care and health provider outcomes: systematic review. Qual Saf Health Care. (2009) 18:181–8. 10.1136/qshc.2007.024232 19467999

[23] Lockley SW Barger LK Ayas NT Rothschild JM Czeisler CA. Effects of health care provider work hours and sleep deprivation on safety and performance. Jt Comm J Qual Patient Saf . (2007) 33(11 Suppl.):7–18. 10.1016/S1553-7250(07)33109-7 18173162

[24] Rubin R Orris P Lau SL Hryhorczuk DO Furner S Letz R. Neurobehavioral effects of the on-call experience in housestaff physicians. J Occup Med Off Publ Ind Med Assoc. (1991) 33:13–8. 1995796 10.1097/00043764-199101000-00007

[25] Lingenfelser T Kaschel R Weber A Zaiser-Kaschel H Jakober B Küper J. Young hospital doctors after night duty: their task-specific cognitive status and emotional condition. Med Educ. (1994) 28:566–72. 7862021 10.1111/j.1365-2923.1994.tb02737.x

[26] Grantcharov TP Bardram L Peter F-J Rosenberg J. Laparoscopic performance after one night on-call in a surgical department: prospective study. BMJ. (2001) 323:1222. 10.1136/bmj.323.7323.1222 11719413 PMC59995

[27] Saxena AD George CFP. Sleep and motor performance in on-call internal medicine residents. Sleep. (2005) 28:1386–91. 10.1093/sleep/28.11.1386 16335328

[28] Veasey S Rosen R Barzansky B Rosen I Owens J. Sleep loss and fatigue in residency training: a reappraisal. JAMA. (2002) 288:1116–24. 10.1001/jama.288.9.1116 12204082

[29] Wali SO Qutah K Abushanab L Basamh R Abushanab J Krayem A. Effect of on-call-related sleep deprivation on physicians' mood and alertness. Ann Thorac Med. (2013) 8:22. 10.4103/1817-1737.105715 23439930 PMC3573553

[30] Tucker P Bejerot E Kecklund G Aronsson G Åkerstedt T. The impact of work time control on physicians' sleep and well-being. Appl Ergon. (2015) 47:109–16. 10.1016/j.apergo.2014.09.001 25479980

[31] Dowell AC Hamilton S McLeod DK. Job satisfaction, psychological morbidity and job stress among New Zealand general practitioners. N Z Med J. (2000) 113:269–72. http://www.ncbi.nlm.nih.gov/pubmed/10935564. 10935564

[32] Chong A Killeen O Clarke T. Work-related stress among paediatric non-consultant hospital doctors. Ir Med J. (2004) 97:203–5. 15490996

[33] Alhifzi S Al-Ghonimy A Aboudi M Al Abdullah R Al Olaish A BaHammam AS. Assessment of sleep quality, daytime sleepiness, and depression among emergency physicians working in shifts. J Nat Sci Med. (2018) 1:17–21. 10.4103/JNSM.JNSM_8_18

[34] Shanafelt TD Bradley KA Wipf JE Back AL. Burnout and self-reported patient care in an internal medicine residency program. Ann Intern Med. (2002) 136:358–67. 10.7326/0003-4819-136-5-200203050-00008 11874308

[35] Mastenbroek NJJM Jaarsma ADC Demerouti E Muijtjens AMM Scherpbier AJJA van Beukelen P. Burnout and engagement, and its predictors in young veterinary professionals: the influence of gender. Vet Rec. (2014) 174:144. 10.1136/vr.101762 24306199

[36] Hatch PH Winefield HR Christie BA Lievaart JJ. Workplace stress, mental health, and burnout of veterinarians in Australia. Aust Vet J. (2011) 89:460–8. 10.1111/j.1751-0813.2011.00833.x 22008127

[37] Ziebertz CM van Hooff MLM Beckers DGJ Hooftman WE Kompier MAJ Geurts SAE. The relationship of on-call work with fatigue, work-home interference, and perceived performance difficulties. Biomed Res Int. (2015) 2015:643413. 10.1155/2015/643413 26558276 PMC4628979

[38] Emmett BM Dovey SM Wheeler BJ. After-hours on-call: the effect on paediatricians' spouses and families. J Paediatr Child Health. (2013) 49:246–50. 10.1111/jpc.12108 23414341

[39] Rout U. Stress among general practitioners and their spouses: a qualitative study. Br J Gen Pract. (1996) 46:157–60. 8731621 PMC1239574

[40] Geurts SAE Sonnentag S. Recovery as an explanatory mechanism in the relation between acute stress reactions and chronic health impairment. Scand J Work Environ Health. (2006) 32:482–92. 10.5271/sjweh.1053 17173204

[41] Meijman TF. Psychological aspects of workload. New Handb Work Organ Psychol. (1998) 2:5–34.

[42] Xanthopoulou D Bakker AB Oerlemans WGM Koszucka M. Need for recovery after emotional labor: differential effects of daily deep and surface acting. J Organ Behav. (2018) 39:481–94. 10.1002/job.2245

[43] Kivimäki M Leino-Arjas P Kaila-Kangas L Luukkonen R Vahtera J Elovainio M. Is incomplete recovery from work a risk marker of cardiovascular death? Prospective evidence from industrial employees. Psychosom Med. (2006) 68:402–7. 10.1097/01.psy.0000221285.50314.d3 16738071

[44] Sluiter JK Frings-Dresen MHW Van Der Beek AJ. The relation between neuroendocrine reactivity and recovery from different natures of work, subjective need for recovery, and health status. J Psychosom Res. (2001) 50:29–37. 10.1016/S0022-3999(00)00213-0 11259798

[45] Ciciolla L Luthar SS. Invisible household labor and ramifications for adjustment: mothers as captains of households. Sex Roles. (2019) 81:467–86. 10.1007/s11199-018-1001-x 34177072 PMC8223758

[46] Conroy T. The kids are alright: working women, schedule flexibility and childcare. Reg Stud. (2019) 53:261–71. 10.1080/00343404.2018.1462478

[47] Market Research Statistics: U.S. Veterinarians. Schaumburg, IL: American Veterinary Medical Association (2017).

[48] Nilsson PM Kecklund G Åkerstedt T. Sleep and recovery. In: *Current Perspectives on Job-Stress Recovery*. Vol 7. Research in Occupational Stress and Well-being. Emerald Group Publishing Limited SV - 7 (2009). p. 205–47. Available online at: https://www.emeraldinsight.com/doi/abs/10.1108/S1479–3555(2009)0000007009 (accessed March 7, 2020).

[49] Sonnentag S Fritz C. The recovery experience questionnaire: development and validation of a measure for assessing recuperation and unwinding from work. J Occup Health Psychol. (2007) 12:204–21. 10.1037/1076-8998.12.3.204 17638488

[50] Iso-Ahola SE. The Social Psychology of Leisure and Recreation. Dubuque, IA: W.C. Brown Co. Publishers (1980).

[51] Kossek EE Ruderman MN Braddy PW Hannum KM. Work–nonwork boundary management profiles: a person-centered approach. J Vocat Behav. (2012) 81:112–28. 10.1016/j.jvb.2012.04.003

[52] Drach-Zahavy A Marzuq N. The weekend matters: EXPLORING when and how nurses best recover from work stress. J Adv Nurs. (2013) 69:578–89. 10.1111/j.1365-2648.2012.06033.x 22606992

[53] Rosenfield S. The effects of women ' s employment : personal control and sex differences in mental health author (s): sarah rosenfield source. J Health Soc Behav. (1989) 30:77–91. 10.2307/2136914 2723381

[54] Hooff MLM van Geurts SAE Beckers DGJ Kompier MAJ. Daily recovery from work: the role of activities, effort and pleasure. Work Stress. (2011) 25:55–74. 10.1080/02678373.2011.570941

[55] Sonnentag S. Psychological detachment from work during leisure time: the benefits of mentally disengaging from work. Curr Dir Psychol Sci. (2012) 21:114–8. 10.1177/0963721411434979

[56] Sprajcer M Jay SM Vincent GE Vakulin A Lack L Ferguson SA. How the chance of missing the alarm during an on-call shift affects pre-bed anxiety, sleep and next day cognitive performance. Biol Psychol. (2018) 137:133–9. 10.1016/j.biopsycho.2018.07.008 30059707

[57] Robbins J Gottlieb F. Sleep deprivation and cognitive testing in internal medicine house staff. West J Med. (1990) 152:82–6. 2309486 PMC1002278

[58] Landrigan CP Rothschild JM Cronin JW Kaushal R Burdick E Latz JT. Effect of reducing interns' work hours on serious medical errors in intensive care units. N Engl J Med. (2004) 351:1838–48. 10.1056/NEJMoa041406 15509817

[59] Barger LK Cade BE Ayas NT Cronin JW Rosner B Speizer FE. Extended work shifts and the risk of motor vehicle crashes among interns. N Engl J Med. (2005) 352:125–34. 10.1056/NEJMoa041401 15647575

[60] Wilhelm BJ Widmann A Durst W Heine C Otto G. Objective and quantitative analysis of daytime sleepiness in physicians after night duties. Int J Psychophysiol Off J Int Organ Psychophysiol. (2009) 72:307–13. 10.1016/j.ijpsycho.2009.01.008 19452604

[61] Heponiemi T Kouvonen A Vänskä J Halila H Sinervo T Kivimäki M. Effects of active on-call hours on physicians' turnover intentions and well-being. Scand J Work Environ Health. (2008) 34:356–63. 10.5271/sjweh.1278 18853067

[62] David P. French Robert K. McKinley AH. GP stress and patient dissatisfaction with nights on call: an exploratory study - GP stress and patient satisfaction. Scand J Prim Health Care. (2001) 19:170–3. 10.1080/028134301316982397 11697558

[63] Sutherland VJ Cooper CL. Job stress, satisfaction, and mental health among general practitioners before and after introduction of new contract. BMJ. (1992) 304:1545. 10.1136/bmj.304.6841.1545 1628056 PMC1882446

[64] Grosch JW Caruso CC Rosa RR Sauter SL. Long hours of work in the U.S.: associations with demographic and organizational characteristics, psychosocial working conditions, and health. Am J Ind Med. (2006) 49:943–52. 10.1002/ajim.20388 17036350

[65] Nitsche N Grunow D. Housework over the course of relationships: gender ideology, resources, and the division of housework from a growth curve perspective. Adv Life Course Res. (2016) 29:80–94. 10.1016/j.alcr.2016.02.001

[66] Sonnentag S Binnewies C Mojza EJ. Staying well and engaged when demands are high: the role of psychological detachment. J Appl Psychol. (2010) 95:965–76. 10.1037/a0020032 20718528

[67] Miller K Birkholt M Scott C Stage C. Empathy and burnout in human service work: an extension of a communication model. Communic Res. (1995) 22:123–47. 10.1177/009365095022002001

[68] Maslach C Jackson SE. Burnout in organizational settings. Appl Soc Psychol Annu. (1984) 5:133–53.

[69] Volk JO Schimmack U Strand EB Lord LK Siren CW. Executive summary of the Merck Animal Health Veterinary Wellbeing Study. J Am Vet Med Assoc. (2018) 252:1231–8. 10.2460/javma.252.10.1231 29701527

[70] Spitznagel MB Ben-Porath YS Rishniw M Kogan LR Carlson MD. Development and validation of a Burden Transfer Inventory for predicting veterinarian stress related to client behavior. J Am Vet Med Assoc. (2018) 254:133–44. 10.2460/javma.254.1.133 30668296

[71] Tomasi SE Fechter-Leggett ED Edwards NT Reddish AD Crosby AE Nett RJ. Suicide among veterinarians in the United States from 1979 through 2015. J Am Vet Med Assoc. (2019) 254:104–12. 10.2460/javma.254.1.104 30668293 PMC6417412

[72] Nett RJ Witte TK Holzbauer SM Elchos BL Campagnolo ER Musgrave KJ. Risk factors for suicide, attitudes toward mental illness, and practice-related stressors among US veterinarians. J Am Vet Med Assoc. (2015) 247:945–55. 10.2460/javma.247.8.945 26421408

[73] Allison SO Eggleston-Ahearn AM Courtney CJ Hardy Malbrue RA Quammen JK. Implementing wellness in the veterinary workplace. J Am Vet Med Assoc. (2016) 249:879–81. 10.2460/javma.249.8.879 27700276

[74] Elkins A Kearney M. Professional burnout among female veterinarians in the United States. J Am Vet Med Assoc. (1992) 200:604–8. 1568895

[75] Wu Y-F Wang P-C Chen Y-C. Gender Differences and Work-Family Conflicts among Emergency Physicians with Intention to Leave. Emerg Med Int. (2018) 2018:3919147. 10.1155/2018/3919147 30510802 PMC6231391

[76] Mitchell DA. Generation Z–striking the balance: healthy doctors for a healthy community. Aust Fam Physician. (2008) 37:665–7. 18704218

[77] Platt B Hawton K Simkin S Mellanby RJ. Suicidal behaviour and psychosocial problems in veterinary surgeons: a systematic review. Soc Psychiatry Psychiatr Epidemiol. (2012) 47:223–40. 10.1007/s00127-010-0328-6 21181111

[78] Scotney RL McLaughlin D Keates HL. A systematic review of the effects of euthanasia and occupational stress in personnel working with animals in animal shelters, veterinary clinics, and biomedical research facilities. J Am Vet Med Assoc. (2015) 247:1121–30. 10.2460/javma.247.10.1121 26517615

[79] Oxley JA Montrose VT Kogan L. E-mental health and the veterinary profession. J Am Vet Med Assoc. (2017) 250:1226–7. 10.2460/javma.250.11.1226 28509633

[80] Yuan X Zhu C Wang M Mo F Du W Ma X. Night shift work increases the risks of multiple primary cancers in women: a systematic review and meta-analysis of 61 articles. Cancer Epidemiol Biomarkers Prev. (2018) 27:25–40. 10.1158/1055-9965.EPI-17-0221 29311165

