# Supplementary material for: On-Call Duties: The Perceived Impact on Veterinarians' Job Satisfaction, Well-Being and Personal Relationships
Source: Front Vet Sci. 2021 Oct 27;8:740852. doi: 10.3389/fvets.2021.740852 (PMC8578875; doi:10.3389/fvets.2021.740852)
Supplement: Supplementary file 1 [file Table_1.DOCX]

# Veterinarians and 'on call' shifts

## Please answer the following questions about you and where you work:

#### 1) What type of practice do you primarily work in?*

( ) Small animal general practice

( ) Small animal emergency practice

( ) Small animal specialty/referral

( ) Mixed animal general practice

( ) Exotics/avian/pocket pets

( ) Equine exclusive

( ) Large animal

( ) Non-clinical veterinary practice (ie, research)

( ) Retired/unemployed

( ) Other: _________________________________________________

#### 2) How would you classify your position?

( ) Full time - more than 40 hours a week

( ) Part-time - less than 40 hours a week

#### 3) Are you an associate or an owner/partner?

( ) Associate

( ) Owner/partner

#### 4) Do you work in an emergency clinic?

( ) Yes

( ) No

#### 5) Years in practice:

( ) 0-5

( ) 6-10

( ) 11-15

( ) 16-20

( ) 20-30

( ) >30

#### 6) Gender:

( ) Male

( ) Female

( ) Other/NA

#### 7) What is your age?

( ) < 30 years

( ) 31-35 years

( ) 36-40 years

( ) 41-45 years

( ) 46-50 years

( ) 51-60 years

( ) 61-70 years

( ) >70 years

#### 8) Marital/partner status:

( ) Married/partnered

( ) Single

( ) Divorced

( ) Other:: _________________________________________________

#### 9) Number of children living at home with you:*

### 10) Ages of children living at home with you: 11) Have you ever had on-call responsibilities?*

( ) Yes

( ) No

#### 12) Do you currently have on-call responsibilities?*

( ) Yes

( ) No

#### 13) Did the fact that your past job entailed being on-call influence your decision to leave?

( ) Yes, it was an important factor in my decision

( ) Yes, it was a moderately important factor in my decision

( ) Yes, but it was only a minimally important factor in my decision

( ) It was not a factor in my decision

#### 14) Did the fact that your current job does not entail being on-call influence your decision to accept the position?

( ) Yes, it was an important factor in my decision

( ) Yes, it was a moderately important factor in my decision

( ) Yes, but it was only a minimally important factor in my decision

( ) It was not a factor in my decision

## On-call responsibilities

#### 15) How large of a role do you feel on call responsibilities would impact a future job choice for you?

( ) On call responsibilities would play no role in any future job decision

( ) On call responsibilities would play a minimal role in any future job decision

( ) On call responsibilities would play a moderate role in any future job decision

( ) On call responsibilities would play a major role in any future job decision

( ) Add NA/other (please explain:):

#### 16) Please indicate the frequency of your on call shifts - average number of nights per month:

( ) 1-4

( ) 5-8

( ) 9-12

( ) 13-16

( ) 17-20

( ) 21-24

( ) 25 or more

#### 17) Please indicate your typical on call schedule:

( ) 1-2 days in a row

( ) 3-4 days in a row

( ) 5-6 days in a row

( ) 7 - 30 days in a row

( ) More than 30 days in a row

#### 18) How often do you have on call responsibilities over the weekend?*

( ) Never

( ) Rarely

( ) Sometimes

( ) Often

( ) Always

#### 19) Please indicate the impact being on call over the weekend has on your weekend?

( ) Extremely negative impact

( ) moderately negative impact

( ) No impact

( ) Moderate positive impact

( ) Extremely positive impact

#### 20) How often do you have on call responsibilities over holidays?*

( ) Never

( ) Rarely

( ) Sometimes

( ) Often

( ) Always

#### 21) Please indicate the impact being on call over the holidays has on your holidays:

( ) Extremely negative impact

( ) moderately negative impact

( ) No impact

( ) Moderate positive impact

( ) Extremely positive impact

#### 22) Do you feel one/some seasons are harder to be on call for than other seasons?

( ) No, not really

( ) Yes, please explain:: _________________________________________________

#### 23) When you are on call, from whom do you receive calls (check all that apply):

[ ] Clients directly

[ ] Answering service

[ ] Another veterinarian

[ ] Veterinary staff members (not veterinarian)

[ ] Other (please explain): _________________________________________________

#### 24) Do you get paid for being on call and available by phone even if you are not called?*

( ) Yes

( ) No

#### 25) Do you get paid extra for being on call if you are called in to take care of patient(s)?*

( ) Yes

( ) No

#### 26) What best describes the extra pay you receive when called in to care for patients?

( ) Flat fee regardless of the number of cases

( ) Based on number of cases

( ) Based on procedures performed

( ) Other: _________________________________________________

#### 27) When you are on call, what percent of nights are you called (but do not go in)?

( ) 1-10%

( ) 11-25%

( ) 26-50%

( ) 51-75%

( ) >75%

#### 28) When you are on call, what percent of nights do you need to actually go in to handle a case?

( ) 1-10%

( ) 11-25%

( ) 26-50%

( ) 51-75%

( ) >75%

#### 29) If you are at work at night taking care of emergency cases, do you get the next day off work?

( ) Yes

( ) No

( ) Depends on the next day’s schedule

( ) Other: _________________________________________________

#### 30) Please rate your level of agreement with the following questions.

|  | **Strongly disagree** | **Disagree** | **Neutral** | **Agree** | **Strongly agree** | **NA** |
| --- | --- | --- | --- | --- | --- | --- |
| I feel the extra pay I get from being on call makes the inconvenience worth it | ( ) | ( ) | ( ) | ( ) | ( ) | ( ) |
| I feel that having on call duties negatively impacts my job satisfaction | ( ) | ( ) | ( ) | ( ) | ( ) | ( ) |
| I feel that it is important to personally be on call in order to take care of my clients and patients | ( ) | ( ) | ( ) | ( ) | ( ) | ( ) |
| I enjoy being on call and helping people during difficult times | ( ) | ( ) | ( ) | ( ) | ( ) | ( ) |
| Overall, I enjoy the nature of the cases that I see on call | ( ) | ( ) | ( ) | ( ) | ( ) | ( ) |
| I experience a great deal of anxiety while on call, regardless of whether I am called in or not | ( ) | ( ) | ( ) | ( ) | ( ) | ( ) |
| I feel being on call causes significant disruptions in my family/personal life | ( ) | ( ) | ( ) | ( ) | ( ) | ( ) |
| I feel I have adequate technical assistance when I see emergency patients while on call | ( ) | ( ) | ( ) | ( ) | ( ) | ( ) |
| I feel I experience negative physical health effects from being on call | ( ) | ( ) | ( ) | ( ) | ( ) | ( ) |
| I feel I experience negative psychological health effects from being on call | ( ) | ( ) | ( ) | ( ) | ( ) | ( ) |
| There are times I worry about my personal safety when I go in to see emergency on call cases | ( ) | ( ) | ( ) | ( ) | ( ) | ( ) |
| I feel unable to relax when I am on call | ( ) | ( ) | ( ) | ( ) | ( ) | ( ) |
| I feel being on call only minimally alters my recreational plans | ( ) | ( ) | ( ) | ( ) | ( ) | ( ) |
| I have difficulty sleeping during nights I am on call even when I am not called | ( ) | ( ) | ( ) | ( ) | ( ) | ( ) |
| I feel being on call is just part of the my job | ( ) | ( ) | ( ) | ( ) | ( ) | ( ) |
| I feel being on call negatively impacts my relationship with my partner | ( ) | ( ) | ( ) | ( ) | ( ) | ( ) |
| I feel being on call negatively impacts my relationship with my children | ( ) | ( ) | ( ) | ( ) | ( ) | ( ) |
| I would take a job for less money if it did not include on call duties | ( ) | ( ) | ( ) | ( ) | ( ) | ( ) |
| I have thought about leaving my current job due to my on call responsibilities | ( ) | ( ) | ( ) | ( ) | ( ) | ( ) |
| I have thought about leaving the profession due to my on call responsibilities | ( ) | ( ) | ( ) | ( ) | ( ) | ( ) |

### 31) Is there anything about being on call (positive or negative) that we have not asked or other general comments?

## Thank You!
